# Harry Potter and personality assessment: The utility of the Sorting Hat Quiz in personality traits’ assessment

**DOI:** 10.1371/journal.pone.0336123

**Published:** 2025-11-24

**Authors:** Lidia Baran, Maria Flakus, Franciszek Stefanek

**Affiliations:** 1 Institute of Psychology, University of Wrocław, Wrocław, Poland; 2 Meta-Research Center, University of Wrocław, Wrocław, Poland; 3 Institute of Philosophy and Sociology, Polish Academy of Sciences, Warszawa, Poland; 4 Institute of Psychology, University of Silesia in Katowice, Katowice, Poland; University of Padova, ITALY

## Abstract

Interactions with fiction influence our behavior and identification, as fiction encompasses elements of day-to-day reality. For instance, *Harry Potter* (HP) readers often identify with one of the Hogwarts Houses, recognizing their personality traits as similar to one of the house’s members. This tendency constitutes a fertile ground for the popularity of the *Sorting Hat Quiz* (SHQ), i.e., a quiz allowing a placement to one of the houses. Recent studies showed that SHQ predicts some personality traits, i.e., Big Five, Dark Triad, and need for cognition. However, those studies were focused only on HP fans. Thus, the presented study aimed to replicate and extend previous studies by (1) using more complex measurements of personality traits and (2) analyzing the obtained results in groups of HP readers and non-readers. Our findings suggested that although participants sorted (or desired to be sorted) into a particular house shared some of the hypothesized personality features, the predictive power of the SHQ was limited only to HP readers, with minor or non-existent effects in the non-reader subsample. We discuss the obtained result in light of the narrative collective assimilation hypothesis and offer directions for future studies focused on the culture-based phenomenon.

## Introduction

From an evolutionary perspective, storytelling is one of the essential human behaviors. The most vivid manifestation of the importance of stories is the prehistoric rock paintings discovered in many parts of the world [1]. Nowadays, storytelling is still a vital part of humans’ everyday life, manifesting, for example, in the growing popularity of numerous fictional universes, e.g., the world of wizards in the *Harry Potter* (HP) universe, Middle Earth in the “Lord of the Rings” [2], or the world of superheroes in Marvel’s movies and comic books [3]. The interest in such stories is not surprising as storytelling serves to fulfill many psychological needs, e.g., developing beliefs and values [4,5], stimulating and explaining the personal and social world, or supporting the understanding of social and internal states and processes [6,7]. Participating in storytelling can create an experience of immersion in its narrative [8,9], which includes, among others, identifying with characters, imagined participation in a story world, and implementing the behavior of the characters in real life [10].

**Fig 1:**
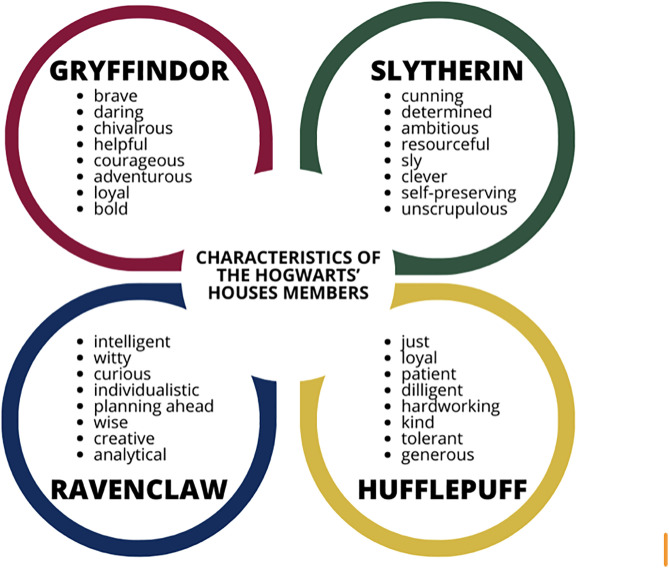
Psychological traits characteristic for members of each of the Hogwarts Houses.

A phenomenon related to this type of immersion is completing tests related to fictional story worlds, i.e., quizzes taken by the fans of certain fictional universes to see, for instance, which character/society they are similar to [11,12]. The most widely empirically analyzed example of such quizzes is the *Sorting Hat Quiz* (SHQ), available at https://www.harrypotter.com SHQ is a short quiz inspired by the magical hat of the same name in the HP books. It consists of ten questions about personal preferences or possible behaviors in described situations. The result obtained after answering them is the name of one of the houses (a kind of fraternity, i.e., Slytherin, Gryffindor, Hufflepuff, Ravenclaw) to which the respondent would belong in the fictional Hogwarts School of Witchcraft and Wizardry. The houses’ specific features are presented in Fig 1.

The SHQ caught the attention of researchers who wanted to determine if its results could differentiate people based on the traits and characteristics of Hogwarts House members. To test this, previous studies compared personality traits, important values, need for cognition, and need to belong among people sorted to each of the Hogwarts Houses [13,14]. Results of Crysel et al. [13] show that compared to other houses, participants sorted as (1) Hufflepuffs were more agreeable and had marginally higher need to belong, (2) Slytherins were more Machiavellian, narcissistic, and psychopathic, (3) Ravenclaws had higher need for cognition, and (4) Gryffindors were marginally more extroverted. The same results, apart from the need to belong for Hufflepuffs, were obtained when comparing participants’ traits based on their wishes regarding the result of sorting. The authors understand those results as a manifestation of synergy between real-life personality traits and their simplified typologies used in narratives. They claim that readers and viewers of the HP series are inclined to engage with the Hogwarts House system as a reflection of distinct characteristics linked with individual personality traits.

A replication of this study by Jacob et al. [14] revealed similarly the highest scores for extroversion among Gryffindors, agreeableness and conscientiousness for Hufflepuffs, Machiavellianism, psychopathy, and narcissism for Slytherins, and intellect for Ravenclaws. Additionally, they found that participants sorted into particular houses differed in important values: conformity for Hufflepuffs, power for Slytherins, and tradition for Gryffindors and Hufflepuffs. Interestingly, similar results were obtained when comparing traits of participants based on their desired sorting results (apart from conscientiousness), but not when comparing traits of participants who were not sorted to the house they desired. Considering the lack of or weak differences between them, the Authors claim that the desire to be assigned to a specific house is a factor that enhances the relationship between house assignment based on the SHQ and personality measures, and therefore drives the actual association between houses and personality.

### The current study

In light of previous research, it seems that a more detailed analysis of the relationship between individual differences and the SHQ results is needed. The Quiz itself is not a psychometric tool based on any psychological theory, and the data administrators do not share any information about its validity or reliability. That is why verifying to what extent the SHQ results can actually differentiate people is the only way to answer the question about what it is measuring.

In the current study, we aimed to extend the results of Crysel et al. [13] and Jakob et al. [14], and replicate Crysel et al.’s [13] study on a Polish sample using methods that allow for more complex measurements of the analyzed traits. In the case of the Dark Triad traits, we decided to use a separate method for each of them rather than three subscales of one method (i.e., the Dark Triad Dirty Dozen in Crysel et al. [13]; the Short Dark Triad in Jakob et al. [14]) to avoid their reported limitations [15] and to verify if previous relationships between them and the house membership will be the same when analyzing facets of psychopathy (i.e., boldness, meanness, and disinhibition) and narcissism (i.e., narcissistic admiration and rivalry). Rather than short (i.e., TIPI in Crysel et al. [13]) or standard (i.e., IPIP 50 in Jakob et al. [14]) measures of the Big Five personality traits, we used the Hexaco model scale, which includes an additional factor of Honesty/Humility describing the tendency not to use manipulation, fight for social status, and break the rules for the personal gain [16]. Finally, to measure the need for cognition, we used the full-length Need for Cognition Scale, which has slightly better psychometric properties than its short form used by Crysel et al. [13].

Based on Crysel et al. [13] and Jakob et al. [14] studies, we hypothesize that people sorted to (1) Gryffindor will have the highest levels of extroversion and openness to experience, (2) Ravenclaw will have the highest need for cognition, (3) Slytherin will have the highest levels of the Dark Triad traits, and (4) Hufflepuffs will have the highest levels of the agreeableness and conscientiousness. Additionally, we expected Hufflepuffs to have the highest levels of honesty/humility and wanted to explore if the high level of the Dark Triad traits for the Slytherins will be replicated when using three separate methods to measure them and will include all facets of psychopathy and narcissism.

Moreover, we wanted to test further the Jakob et al. [14] claim that the association between personality traits and house membership is driven by the desire to be assigned to a specific House. Previous research shows that the content of the narrative may influence how its recipients perceive themselves and behave. For example, watching characters smoking in the movie increases viewers’ intention to smoke if the character is easy to identify with [17]. Such phenomena do not seem to be just behavioral mimicking, but rather, as the narrative collective assimilation hypothesis [18] states, the experience of a narrative may lead people to become a part of the collective described within the story, creating a specific type of group identification underlying a person’s relationship with narratives. As an example of this hypothesis, Gabriel and Young [18] asked participants to read passages from either a book about wizards (HP series) or a book about vampires (Twilight series). The results showed that people who read the HP books saw themselves as psychologically closer to the wizards described in the story, while those who read the Twilight books indicated a closer connection to vampires. It seems then that the assimilation with the narrative may be related to people’s desire to be a part of a specific group, such as a house, and, as a result, seeing oneself as similar to the members of the chosen group.

To test that notion, we verified effects in two groups of participants: (1) respondents who read the books from the HP saga and (2) respondents who had not read any Harry Potter books.. Film adaptations do not fully convey the detailed personality traits and values associated with each Hogwarts House, which are extensively described in the books. Therefore, we expected that the pattern of associations between house membership and personality traits would be qualitatively different among book readers, for whom these characterizations were explicit. Verifying those associations in two groups will allow us to explore the differences in traits between particular houses might be related to previous assimilation with the narrative. Secondly, we analyzed differences in psychological traits between both sorted and desired house membership to see if any of them may be related to already existing desires to be a member of a chosen house. Finally, we analyzed differences in psychological traits between house membership among people who were not sorted to the house they desired and among people who desired to be sorted to a house different from the sorting result. This way, we could verify if the sorting test results differentiate participants on the psychological traits level, even if they are opposite to their desires, or if the differentiating factor is related instead to an individual desire to be a member of a particular house.

Even though the SHQ is not a psychometrically verified method, studying it seems useful for two reasons. First, exploring the test itself and broadening the knowledge about its relationships with other psychological constructs can be useful to the people who use it and also to researchers studying other concepts related to storytelling, such as the associations between identification with a character and personality traits like agreeableness [19], or identification with the group and job preferences [20]. This aim might be especially important in light of already noticed misconceptions about the utility of using cultural phenomena in diagnosis or predictions [21]. Second, the growing interest in incorporating pop-cultural elements in psychological practice (e.g., [22,23]) makes exploring possible relationships between identification with certain characters or groups and individual traits important. Understanding these connections might be helpful to practitioners who wish to use client/patient cultural experiences as a part of professional interventions.

## Methods

### Participants and procedure

The study was approved by the University of Silesia in Katowice Ethics Committee. Participants were recruited using social media (Facebook fan pages and groups) and completed questionnaires anonymously through the LimeSurvey online platform (data collection start: September 25, 2020; end: December 10, 2020). The first screen contained a detailed description of the study and participation requirements, followed by a written consent to participate (i.e., a checkbox with consent). Next, participants answered general questions on gender, age, education, and fluency in English, and questions related to HP fanship (i.e., reading books, watching movies, SHQ results) and filled out the questionnaires in the order presented below. After answering all questions, participants could opt to receive information about the general results of the study and/or to participate in the prize drawing (four gift vouchers worth 50 PLN/ ~ 12 USD). The presented study is part of a larger data collection investigating the relationship between personality traits, work ethics, and popular culture.

We analyzed data only from participants who were 18 years or older (i.e., minor participants were excluded from analyses). Also, we excluded incomplete surveys from the data analysis (*n* = 2164). This resulted in a total sample size of 677 participants aged from 18 to 55 years (*M* = 22.80, *SD* = 4.49), predominantly women (*n* = 551, 81.39%), with 2.22% non-binary participants (*n* = 15). All participants were residents of Poland or spoke Polish fluently.

The total sample was divided into two subsamples. The first of them comprised 578 HP books readers (age: *M* = 22.83, *SD* = 4.45, range: 18−55; mostly women: *n* = 480, 83.05%) and, therefore, were familiar with explicit descriptions of four Hogwarts Houses. The second group comprised 99 participants who had not read HP books (age: *M* = 22.62, *SD* = 4.77, range: 18−49; mostly women: *n* = 71, 71.72%) and, consistently, were not familiar with the explicit description of four Hogwarts Houses. Yet, most participants in sample 2 had seen the HP movies (*n* = 91, 91.92%; in contrast, almost all readers had also seen the movies: *n* = 575, 99.5%), so they had some general orientation regarding the universe created by J.K. Rowling. However, there were statistically significant differences between groups in the level of liking both the movies (U Mann-Whitney’s test: *U* = 16290.00, *p* < .001, *r*_*bs*_ = −.47) and the franchise (*U* = 11096.00, *p* < .001, *r*_*bs*_ = −.61). Consistently, HP readers liked the movies (*M* = 7.83, *SD* = 2.16, median = 8.00) and franchise (*M* = 6.44, *SD* = 2.91, median = 7.00) to a greater degree than participants who have only seen the HP movies (movies: *M* = 5.28, *SD* = 3.23, median = 6.00; franchise: *M* = 2.66, *SD* = 3.09, median = 1.00). Additional information about all participants and both groups concerning *Harry Potter* fanship are available in S1 and S2 Tables.

### Measures

To assess experiences related to the *Harry Potter* universe, we asked participants: 1) if they read any of the HP books (yes/no), how many times (inserting number), and are they fans of the books (0 = not at all, 10 = to a great degree); 2), if they watched any of the HP movies (yes/no), how many times (inserting number), and if they are fans of the movies (0 = not at all, 10 = to a great degree); 3), are they fans of HP extended universe – books and movies besides the main series, Pottermore or MuggleNet platforms (0 = not at all, 10 = to a great degree). Next, we instructed participants to visit the website https://www.harrypotter.com/ and either create or log in to their accounts, take the Sorting Hat Quiz, or check their previous results (we did not inquire about the exact time of its finalization, though), and then go back to the questionnaire and choose from the list which of the houses they were sorted into. Finally, we also asked them to choose from the list which house they wanted to be sorted into.

Need for cognition was measured with the Polish adaptation [24] of the Need for Cognition Scale [25], consisting of 36 items (i.e., “I’d rather learn how to solve a problem than get a ready-made solution”), which participants rated using a five-point scale (1 = strongly disagree, 5 = strongly agree). The index of need for cognition was calculated as a sum of all the answers (Cronbach’s α = .91).

Narcissism was assessed using the Polish adaptation [26] of the Narcissistic Admiration and Rivalry Questionnaire [27]. The questionnaire comprises of 18 items measuring two facets of narcissism: agentic admiration (9 items, i.e., “I am great,” α = .85) and antagonistic rivalry (9 items, i.e., “I want my rivals to fail,” α = .85). The items were rated on a six-point Likert-type scale (1 = not agree at all, 6 = agree completely) and averaged to create indices for both facets.

Machiavellianism was measured with the MACH-IV ( [28]; Polish translation: [29]), which comprises 20 items (i.e., “The best way to handle people is to tell them what they want to hear”) rated on a seven-point Likert-type scale (1 = completely disagree, 7 = completely agree). The index of Machiavellianism was calculated as a sum of all the answers (α = .84).

To assess psychopathy, we used the shortened Polish version of the Triarchic Psychopathy Measure [30]– TriPM-41 [31]. Participants rated statements on a four-point scale (0 = completely false; 3 = completely true). Items were summed to create indices for the three subscales: Meanness (10 items, e.g., “I sympathize with others’ problems”; α = .92), Disinhibition (16 items, e.g., “I often get bored quickly and lose interest”; α = .84), and Boldness (15 items, e.g., “I have a knack for influencing people”; α = .85).

HEXACO personality traits [32] were measured with the Polish Personality Lexicon [33]. Participants assessed on a five-point scale (1 = definitely no; 5 = definitely yes) the degree to which 180 adjectives describe them. Scores were summed to create indices for honesty-humility (i.e., cunning, unselfish; α = .92), emotional stability (In the original Szarota et al.‘s [33] work, as well as in the HEXACO taxonomy [32] to describe this factor, authors used the term “emotionality”. However, the results of Szarota et al. suggested a reverse scoring of items related to a lower emotionality, i.e., higher emotional stability. Thus, we decided to rename this factor to “emotional stability” in order to provide readers with a more intuitive and straight-forward interpretation of the study’s results.) (i.e., faint-hearted, fearless; α = .82), extroversion (i.e., active, brisk; α = .93), agreeableness (i.e., impulsive, gentle; α = .93), conscientiousness (i.e., thoughtful, careful; α = .95), and openness to experience (i.e., gifted, creative; α = .93).

## Results

Descriptive statistics for Harry Potter readers and non-readers are reported in Table 1.

**Table 1:** Descriptive statistics for all variables.

		*Harry Potter* readers (subsample 1)				*Harry Potter* non-readers (subsample 2)			
		Sorted		Desired		Sorted		Desired	
		*M*	*SD*	*M*	*SD*	*M*	*SD*	*M*	*SD*
***HEXACO personality traits***									
Conscientiousness	Hufflepuff	107.87	20.12	103.27	24.22	113.11	22.59	108.31	22.10
	Others	110.65	21.06	111.15	20.24	110.82	22.31	112.05	22.41
Extroversion	Gryffindor	97.50	20.22	100.98	20.21	102.72	19.87	101.69	20.98
	Others	94.45	20.88	92.79	20.52	93.89	20.78	92.50	20.06
Agreeableness	Hufflepuff	109.44	17.28	107.24	18.24	104.78	20.07	99.06	19.83
	Others	99.99	21.22	100.80	21.14	99.43	20.08	101.24	20.28
Emotional stability	Hufflepuff	85.43	13.03	82.61	12.78	85.15	14.53	83.50	14.10
	Others	91.15	14.73	91.24	14.54	91.39	13.72	90.88	13.92
Honesty/Humility	Hufflepuff	116.77	13.58	115.25	12.57	117.52	14.04	116.88	15.95
	Others	107.37	19.33	108.08	19.36	109.67	17.24	110.83	16.79
Openness	Gryffindor	115.39	15.12	116.25	16.96	115.56	21.17	111.85	15.92
	Others	116.43	16.65	116.25	14.60	111.95	19.22	113.52	21.89
***Dark Triad personality traits***									
Machiavellianism	Slytherin	88.41	17.22	89.11	17.16	80.21	17.53	19.19	3.69
	Others	74.87	14.92	75.24	15.10	75.91	14.31	12.61	1.49
Narcissistic admiration	Slytherin	3.57	1.04	3.64	1.05	3.27	.92	3.25	.99
	Others	3.15	.90	3.14	.90	3.24	.87	3.24	.83
Narcissistic rivalry	Slytherin	3.43	1.08	3.36	1.05	2.61	1.36	2.87	1.33
	Others	2.59	.87	2.65	.91	2.56	.88	2.46	.79
Psychopathy	Slytherin	51.45	16.66	51.76	16.13	44.79	18.37	44.70	19.81
	Others	41.08	14.34	41.46	14.78	41.85	14.29	41.56	12.97
Psychopathic meanness	Slytherin	10.84	6.85	10.61	6.56	7.53	8.11	8.85	8.10
	Others	6.32	5.86	6.62	6.17	6.54	6.69	5.92	6.25
Psychopathic boldness	Slytherin	23.79	8.07	24.33	8.28	23.21	8.05	20.59	9.94
	Others	20.49	8.39	20.44	8.24	21.50	8.37	22.29	7.61
Psychopathic disinhibition	Slytherin	16.82	8.71	16.81	9.09	14.05	9.96	15.26	10.04
	Others	14.27	8.42	14.40	8.30	13.81	8.18	13.33	7.85
***Need for cognition***									
Need for cognition	Ravenclaw	136.55	16.84	136.66	15.25	113.21	17.91	22.01	5.34
	Others	129.57	19.23	129.29	19.83	130.17	19.78	18.74	2.07

Despite slight inequalities, enough participants were sorted into each house for adequate statistical comparisons (*n*s for readers vs. non-readers: Gryffindor: 133 vs. 25, Ravenclaw: 167 vs. 28, Hufflepuff: 98 vs. 27, Slytherin: 180 vs. 19). Also, regarding desired houses, enough participants chose each house to provide adequate comparisons (*n*s for readers vs. non-readers: Gryffindor: 167 vs. 39, Ravenclaw: 180 vs. 17, Hufflepuff: 71 vs. 16, Slytherin: 160 vs. 27). Most participants were sorted into their chosen house: χ^2^ (9) = 355.91, *p* < .001, Cramer’s *V* = .45.

### Differences in personality traits between sorted houses

To inspect the differences between each house, we applied the Helmert contrast method (Table 2), comparing the results for a particular group to the values obtained by participants sorted to the other three houses (e.g., in the case of conscientiousness: Hufflepuff compared to the average scores reported in Gryffindor, Ravenclaw, and Slytherin). It is an appropriate way to test differences between one focal group with a cluster of other groups, preventing substantial inflation of Type I error rates. The method was applied consistently with Crysel et al. [13].

**Table 2:** Differences between houses (sorted) – Helmert contrasts.

	Comparison	*Harry Potter* readers (subsample 1)				*Harry Potter* non-readers (subsample 2)			
		Contrast				Contrast			
		*t* (574)	*p*	Corrected *p*	Cohen’s *d* [95%CI]	*t* (95)	*p*	Corrected*p*	Cohen’s *d*
***HEXACO personality traits***									
Conscientiousness	H *vs* others	−1.23	.221	.884 (.442)	−.13 [-.35,.09]	.45	.653	1.00 (.694)	.10 [-.34,.55]
Extroversion	G *vs* others	1.89	.060	.180 (.090)	.15 [-.05,.34]	1.78	.078	.180 (.094)	.43 [-.03,.89]
Agreeableness	H *vs* others	4.16	<.001	<.001 (<.001)	.46 [.24,.68]	1.13	.262	.786 (.393)	.27 [-.18,.71]
Emotional stability	H *vs* others	−3.67	<.001	<.001 (<.001)	−.40 [-.61, -.18]	−2.04	.044	.132 (.066)	−.45 [-.89,.00]
Honesty/Humility	H *vs* others	4.56	<.001	<.001 (<.001)	.51 [.29,.73]	2.15	.035	.140 (.070)	.48 [.03,.92]
Openness	G *vs* others	−.15	.880	1.00 (.880)	−.06 [-.26,.13]	.74	.459	1.00 (.880)	.18 [-.27,.64]
***Dark Triad personality traits***									
Machiavellianism	S *vs* others	9.82	<.001	<.001 (<.001)	.86 [.68, 1.05]	1.12	.267	.376 (.267)	.29 [-.22,.79]
Narcissistic admiration	S *vs* others	5.18	<.001	<.001 (<.001)	.44 [.26,.62]	.14	.888	1.00 (.888)	.04 [-.46,.54]
Narcissistic rivalry	S *vs* others	10.20	<.001	<.001 (<.001)	.89 [.71, 1.08]	.24	.813	(.813)	.06 [-.45,.56]
Psychopathy	S *vs* others	7.79	<.001	<.001 (<.001)	.69 [.51,.87]	.75	.458	.692 (.458)	.19 [-.31,.70]
Psychopathic meanness	S *vs* others	8.22	<.001	<.001 (<.001)	.73 [.55,.91]	.54	.594	1.00 (.713)	.14 [-.36,.64]
Psychopathic boldness	S *vs* others	4.69	<.001	<.001 (<.001)	.40 [.22,.58]	.78	.437	1.00 (.655)	.21 [-.30,.71]
Psychopathic disinhibition	S *vs* others	3.27	<.001	.006 (.006)	.30 [.12,.48]	.13	.896	1.00 (.896)	.03 [-.47,.53]
***Need for cognition***									
Need for cognition	R *vs* others	3.97	<.001	<.001 (<.001)	.38 [.19,.56]	.72	.475	.950 (.570)	.22 [-.28,.60]

*Note*. House (sorted; number of participants in groups are provided in parentheses – for readers and non-readers, respectively): G – Gryffindor (133 vs. 25), R – Ravenclaw (167 vs. 28), H – Hufflepuff: (98 vs. 27), S – Slytherin (180 vs. 19). For corrected p-values, Holm–Bonferroni correction was applied, with Benjamini–Hochberg adjusted p-values provided in the parentheses.

In contrast to Crysel et al. [13], to account for multiple comparisons, p-values were adjusted using the Holm–Bonferroni procedure (family-wise error rate control). For transparency, we also report Benjamini–Hochberg adjusted p-values (false discovery rate control). Also, we report 95% confidence intervals for effects observed for each contrast to provide a more precise and accurate assessment of their magnitude. Additionally, it facilitated comparison across groups despite differences in sample size and helps to convey the uncertainty associated with smaller subgroups, and, thus, reduced statistical power.

Among HP readers (subsample 1), Gryffindors were marginally higher in extroversion than the members of other houses, with no difference in openness between Gryffindors and other houses. Also, Helmert’s contrast showed no differences in conscientiousness, comparing Hufflepuffs against other houses. However, Hufflepuffs declared themselves as more agreeable than members of the other houses and presented themselves as more honest/humble and less emotionally stable than other participants. Slytherins presented themselves as the most narcissistic and psychopathic (total score and facets) compared to other participants, and they had the highest scores of Machiavellianism. Finally, participants sorted to Ravenclaw had a higher score of need for cognition than the other houses.

Among HP non-readers (subsample 2), results showed fewer statistically significant differences between the hypothetically dominant houses and respondents assigned to other houses. However, Helmert’s contrast suggested that Gryffindors had a marginally higher extroversion score than the members of other houses. Also, Hufflepuffs tended to be more honest/humble and less emotionally stable than the rest of the respondents. Yet, all differences were non-significant when *p*-values were corrected.

In general, the averages of effect sizes’ absolute values were.46 and.22 for HP readers and non-readers, respectively, suggesting medium effect observed for readers and small for non-readers..

### Differences in personality traits between desired houses

Consistent with the previous analyses, we applied Helmert contrast to inspect the differences in personality trait scores between the desired houses and verify the hypotheses regarding distinguishing features of particular Hogwarts Houses in both subsamples (Table 3).

**Table 3:** Differences between houses (desired) – Helmert contrasts.

	*Comparison*	*Harry Potter readers (subsample 1)*				*Harry Potter non-readers (subsample 2)*			
		*t (574)*	*p*	Corrected *p*	*Cohen’s d*	*t (95)*	*p*	Corrected *p*	*Cohen’s d*
***HEXACO personality traits***									
Conscientiousness	H vs others	−2.98	.003	.015 (.009)	−.38 [-.63, -.13]	−.40	.694	1.00 (.694)	−.17 [-.70,.37]
Extroversion	G vs others	5.16	<.001	<.001 (<.001)	.40 [.22,.58]	2.12	.036	.144 (.072)	.45 [.04,.86]
Agreeableness	H vs others	2.59	.010	.050 (.024)	.31 [.06,.56]	−.29	.774	1.00 (.874)	−.11 [-.64,.43]
Emotional stability	H vs others	−4.92	<.001	<.001 (<.001)	−.60 [-.85, -.35]	−1.85	.068	.136 (.082)	−.53 [-1.07,.01]
Honesty/Humility	H vs others	3.40	<.001	.004 (.002)	.38 [.14,.63]	1.37	.173	.346 (.208)	.36 [-.18,.90]
Openness	G vs others	1.43	.154	.770 (.462)	.01 [-.17,.19]	−.24	.813	1.00 (.880)	−.08 [-.49,.32]
**Dark Triad personality traits**									
Machiavellianism	S vs others	9.70	<.001	<.001 (<.001)	.88 [.69, 1.07]	2.24	.027	.092 (.041)	.52 [.07,.97]
Narcissistic admiration	S vs others	6.49	<.001	<.001 (<.001)	.53 [.35,.72]	.23	.816	1.00 (.888)	.02 [-.43,.46]
Narcissistic rivalry	S vs others	8.16	<.001	<.001 (<.001)	.74 [.55,.92]	1.82	.072	.216 (.108)	.43 [-.02,.88]
Psychopathy	S vs others	7.77	<.001	<.001 (<.001)	.68 [.49,.87]	1.29	.200	.600 (.300)	.21 [-.24,.65]
Psychopathic meanness	S vs others	6.92	<.001	<.001 (<.001)	.64 [.45,.82]	2.14	.035	.105 (.053)	.43 [-.02,.87]
Psychopathic boldness	S vs others	5.93	<.001	<.001 (<.001)	.47 [.29,.66]	−.34	.735	1.00 (.735)	−.21 [-.65,.24]
Psychopathic disinhibition	S vs others	2.98	.003	.015 (.009)	.28 [.10,.47]	.86	.390	1.00 (.585)	.23 [-.22,.67]
**Need for cognition**									
Need for cognition	R vs others	4.88	<.001	<.001 (<.001)	.40 [.22,.58]	−.30	.767	.950 (.767)	−.09 [-.61,.43]

Note. House (desired; number of participants in groups are provided in parentheses – for readers and non-readers, respectively): G – Gryffindor (167 vs. 39), R – Ravenclaw (180 vs. 17), H – Hufflepuff: (71 vs. 16), S – Slytherin (160 vs. 27). For corrected p-values, Holm–Bonferroni correction was applied, with Benjamini–Hochberg adjusted p-values provided in the parentheses.

Among HP readers, respondents who aspired to be Gryffindors were marginally higher in extroversion scores than the members of other houses, while the difference in openness was not statistically significant. Also, results showed differences in conscientiousness, agreeableness, emotional stability, and honesty/humility scores while comparing people who wanted to be sorted in Hufflepuff against other houses. Hufflepuffs had higher agreeableness and honesty/humility and lower emotional stability and conscientiousness scores than members of the other houses. Regarding “aspiring” Slytherins, they had higher declared narcissism and psychopathy scores (total score and facets) compared to other participants. They also had higherf Machiavellianism scores. Finally, participants who desired to be Ravenclaws had a higher need for cognition score than the other houses.

Among HP non-readers, results generally showed fewer statistically significant differences between the Hogwarts houses compared to HP readers. Helmert contrast suggested that “aspiring” Gryffindors performed had a higher extroversion score than the members of other houses, with Hufflepuffs tending to have loweremotional stability scores than the rest of the participants. Also, participants who declared they wanted to be in Slytherin had higher scores in Machiavellianism and psychopathic meanness and marginally higher in narcissistic rivalry than the rest of the respondents. Again, all differences were non significant when *p-*value were corrected.

Most results were consistent with those obtained for groups distinguished based on SHQ (see Table 2). In general, the averages of effect size’s absolute values were.48 and.27 for HP readers and non-readers, respectively, suggesting medium effects observed for the latter and small for the formers.

### Differences in personality traits between sorted and desired houses for people who were sorted against their preference

Finally, we applied Helmert contrast to inspect the differences in personality trait scores between participants who were sorted to a house they did not desire (e.g., sorted to Gryffindor but desired to be in a different house) and participants who desired to be in a different house than the one they were sorted to (i.e., desired to be in Gryffindor, but were sorted to different house). In this case, we performed analyses only for HP readers, as in the case of non-readers, the frequencies in each group were insufficient (*n* < 10) in many cases (i.e., for sorting results – Gryffindor: *n* = 11, Ravenclaw: *n* = 17, Hufflepuff: *n* = 16, Slytherin: *n* = 7; for the desired house – Gryffindor: *n* = 25, Ravenclaw: *n* = 6, Hufflepuff: *n* = 5, S – Slytherin: *n* = 15). The obtained results are presented in Table 4.

**Table 4:** Differences in personality traits between sorted and desired houses for people who were sorted against their preference – Helmert contrasts.

	Comparison	*Harry Potter* readers (sorting)				*Harry Potter* readers (desired house)			
		*t* (231)	*p*	Corrected *p*	Cohen’s *d*	*t* (231)	*p*	Corrected *p*	Cohen’s *d*
***HEXACO personality traits***									
Conscientiousness	H *vs* others	−.57	.573	1.00 (.694)	−.09 [-.39,.20]	−3.29	.001	.006 (.006)	−.63 [-1.00, -.25]
Extroversion	G *vs* others	−1.05	.296	.296 (.296)	−.17 [-.49,.14]	3.45	<. 001	.003 (.002)	.46 [.19,.73]
Agreeableness	H *vs* others	2.53	.012	.050 (.024)	.38 [.09,.68]	.16	.874	1.00 (.874)	.03 [-.34,.40]
Emotional stability	H *vs* others	−1.45	.149	.149 (.149)	−.22 [-.51,.08]	−3.40	<.001	.003 (.002)	−.60 [-.38,.36]
Honesty/Humility	H *vs* others	1.83	.068	.204 (.102)	.28 [-.01,.58]	.04	.969	.969 (.969)	−.01 [-.98, -.23]
Openness	G *vs* others	−.26	.795	1.00 (.880)	−.05 [-.36,.27]	1.95	.053	.318 (.318)	.16 [.11,.43]
***Dark Triad personality traits***									
Machiavellianism	S *vs* others	2.28	.023	.092 (.041)	.35 [.05,.64]	1.32	.188	.376 (.226)	.23 [-.12,.57]
Narcissistic admiration	S *vs* others	.56	.577	1.00 (.865)	.07 [-.22,.36]	1.82	.069	.276 (.138)	.22 [-.12,.56]
Narcissistic rivalry	S *vs* others	3.70	<.001	.001 (<.001)	.56 [.26,.86]	−.41	.685	1.00 (.813)	−.05 −.39,.29]
Psychopathy	S *vs* others	1.94	.054	.216 (.108)	.28 [-.01,.58]	0.95	.346	.692 (.415)	.12 [-.22,.46]
Psychopathic meanness	S *vs* others	2.79	.006	.024 (.012)	.43 [.13,.72]	−.15	.879	1.00 (.879)	−.01 [-.35,.33]
Psychopathic boldness	S *vs* others	.38	.708	1.00 (.735)	.04 [-.25,.33]	1.16	.246	.984 (.492)	−.01 [-.24,.44]
Psychopathic disinhibition	S *vs* others	1.15	.250	1.00 (.500)	.17 [-.13,.46]	.68	.500	1.00 (.600)	.13 [-.22,.47]
***Need for cognition***									
Need for cognition	R *vs* others	1.36	.174	.522 (.261)	.20 [-.09,.48]	2.53	.012	.048 (.024)	.28 [.01,.55]

*Note.* House (sorted, but not the one they wanted to be sorted in): G – Gryffindor (*n*  = 49), R – Ravenclaw (*n* = 66), H – Hufflepuff (*n* = 60), S – Slytherin (*n* = 60). Frequencies for desired house (i.e., desired, but not the one they were sorted to): Gryffindor (*n* = 83), Ravenclaw (*n* = 79, Hufflepuff (*n* = 33), Slytherin (*n* = 40). For corrected p-values, Holm–Bonferroni correction was applied, with Benjamini–Hochberg adjusted p-values provided in the parentheses.

Regarding sorted houses, HP readers sorted into Gryffindor against their will did not have higher extroversion or openness scores than the members of other houses. Also, results showed no differences in conscientiousness, emotional stability, and honesty/humility scores when comparing people unwillingly sorted in Hufflepuff against other houses. However, “involuntary” Hufflepuffs had significantly higher agreeableness scores compared to the members of other houses. Regarding “involuntary” Slytherins, they described themselves as more Machiavellian, mean, and narcissistically rival than others, with marginally higher scores of psychopathy. Finally, the difference between “involuntary” Ravenclaws and other participants in need for cognition was not statistically significant.

Regarding desired houses, “aspiring” Gryffindors (sorted against their will into a different house) had higher scores in extroversion and marginally in openness than others. Also, “aspiring” Hufflepuffs had lower scores in conscientiousness and emotional stability than people wanting to be in different houses. Regarding “aspiring” Slytherins, the differences between them and other participants were not statistically significant in the case of all Dark Triad traits. Finally, “aspiring” Ravenclaws had a higher need for cognition score than other participants.

## Discussion

In this study, we examined the associations between various declared personality traits and Sorting Hat Quiz (SHQ) results, a non-psychological example of fandom-based quizzes, which allows assigning a respondent to one of the Harry Potter series’s Hogwarts Houses, i.e., Gryffindors, Slytherin, Ravenclaw, or Hufflepuff. Specifically, we aimed to assess to what extent the SHQ results can differentiate people in self-reported personality traits, such as the Big Five, Dark Triad, and need for cognition. As previous results of Crysel et al. [13] and Jakob et al. [14] suggested differences between houses’ members in those traits, we wanted to replicate Crysel et al. [13] results in the Polish sample and expand the discussion about the possible nature of obtained differences in a twofold manner by (1) using more complex operationalizations of personality traits and (2) analyzing the results in two groups – people who read and did not read books from HP series.

Our results generally confirmed the effects described in previous studies, suggesting that HP readers assigned (i.e., based on the results of SHQ) and wanting to be assigned (i.e., based on their preference) to different Hogwarts Houses varied in certain self-reported personality trait scores. More specifically, Gryffindors declared themselves as marginally more extroverted (i.e., enjoyed social gatherings and situations, allowing them to lead others), and Ravenclaws had higher need for cognition scores (i.e., engaged in and enjoyed activities requiring thinking). Slytherins declared themselves as more (1) Machiavellian (i.e., interested in self-interest and exploitation of others), (2) psychopathic – both in terms of the total score (i.e., reckless and engaging in antisocial, selfish behaviors), and three psychopathy facets: boldness (i.e., tend to be fearless), meanness (i.e., not empathetic), and disinhibition (i.e., highly impulsive), and (3) narcissistic in two facets of grandiose narcissism: admiration (i.e., being charming towards others to receive their admiration) and rivalry (i.e., being aggressive in situations threatening their ego). Lastly, Hufflepuffs answers imply that they see themselves as more agreeable (i.e., tend to look for compromises and control their temper) and honest/humble (i.e., show a low tendency to break rules or seek entitlement). We only partially replicated differences in declared conscientiousness, which, according to previous research by Crysel et al. [13] and Jakob et al. [14], should be higher among Hufflepuffs – we found this effect only in case of assignments made based on respondents’ preference, but not based on actual SHQ results. However, contrary to previous research, we found that Hufflepuffs answered less emotionally stable (i.e., were prone to feel worry in stressful situations and need emotional support from others) compared to other respondents – we found this effect both in groups derived based on respondents’ preference and actual SHQ results.

Interestingly, among individuals who had not read the HP series, most of the previously noted differences did not emerge. In this group, we observed only a few effects: ‘aspiring’ and ‘actual’ Gryffindors tended to score marginally higher on extroversion, while Hufflepuffs scored less on emotional stability and scored higher on honesty–humility. The latter effect was observed only among ‘actual’ Hufflepuffs. Also, “aspiring” Slytherins had higher scores in Dark Triad traits. Yet, the difference was absent for “actual” Slytherins.

In line with previous studies by Crysel et al. [13] and Jakob et al. [14], our results support the notion that the SHQ can differentiate people’s personality trait scores to a small level, as people generally tend to use fiction to learn and extend their knowledge and awareness about themselves and their social groups [18,34]. It also supports the idea that fictional aspects of pop-culture phenomena, such as the HP series, may at least on a small level reflect well-established details of everyday psychological reality. Hence, although Hogwarts Houses are imaginary and non-existent, individual differences related to them are present in readers and around them in everyday life. For instance, we probably all know some people interested in the exploitation of others (Slytherins), those who enjoy cognitive activities (Ravenclaws), people who like leading others (Gryffindors), and those who tend to look for compromises (Hufflepuffs). As such human portraits may seem somehow stereotyped, they vividly and realistically describe the common experiences of people, which in turn fosters the identification processes [35].

Nevertheless, our results also show that the so-called “power” of the SHQ (and probably other fandom-based quizzes) can be predicted by a quiz-participants’ knowledge of the “laws” that rule the fictional universe. In our case, without the awareness of which traits are related to each Hogwart House, either SHQ has limited ability to sort respondents to an “appropriate” house, or they seem to demonstrate limited ability to identify themselves with a particular house validly. It may show that, in line with some studies [20], fandom-based quizzes have limited predictive power in areas of personality and behavior, i.e., when people are not immersed in fiction. This provides evidence towards the narrative collective assimilation hypothesis [18], suggesting that remembering a narrative may affect personality self-perception, which may lead to answering questions in SHQ and personality tests in line with characteristics of particular house members, which is not or, to a lesser degree, possible for people who did not read books but watched the HP movies. That is why it seems understandable that the obtained differences in the non-readers’ group were related to Gryffindor, to which three main movie characters belong, and to Hufflepuff, which members stand out most.

However, remembering a narrative may also influence self-perception when fragments of the narrative are not engaged in, as the SHQ does not directly provide any reference to the parts of the HP series. Hence, we propose that intensive knowledge of the fictional universe and anticipation of measurement purpose (e.g., personality) possibly evokes enough memories of the narrative to make it possible to engage in the previously assimilated narrative once more, e.g., describe themselves in its terms as a manifestation of collective assimilation (i.e., perceiving oneself as closer to the group that the person esteems or favors). However, our results also imply that tests developed to measure psychological traits can be less valid when they are used in the context of a certain narrative [36–38] because it is not clear whether they remain independent from narrative collective assimilation evoked by being reminded of the narrative associated with the assessment.

Our results may also demonstrate the significance of the strength of the relationship between fiction and its consumers. Research has shown that building a long-lasting fiction-consumer relationship may foster an identification process [39]. Regarding HP, this relationship was built over more than 14 years for some individuals. For many, this series was an essential part of self-growth and maturation, which may boost their identification with the series. Hence, unsurprisingly, for many people, four Hogwarts Houses may be a certain representations of individual differencess, for instance, as the Big Five or Dark Triad are for personality psychologists.

Regarding the differences between people who desired to be in a certain house, our results indicate that, as Jakob et al. [24] claimed, the association between psychological traits and house membership may be driven by the desire to be assigned to a specific house. This motivation, in turn, might result from participants’ high self-knowledge about the actual, observed similarity between their psychological traits and characteristics of a particular house or their wish to be a member of a specific house even if their psychological characteristics are not typical for this particular group. As a result, the obtained differences for sorted and desired house memberships might indicate that the self-knowledge of the participants is a more reliable predictor of their psychological characteristics than the SHQ results or that their responses in personality scales were modified by their ideas about members of desired house activated by questions about sorted and desired house membership.

Apart from replicating the previous results of Crysel et al. [13], we wanted to expand their perspective further. Thus, we examined differences in psychological traits between participants who were not sorted to the house they desired and between participants who desired to be in a different house than the one they were sorted to. Among HP readers, undesired Hufflepuffs tended to declare themselves as more agreeable, and Slytherins more Machiavellianistic, mean, and narcissistically rivalious. On the other hand, desired Gryffindors declared themselves as more extraverted, Hufflepuffs less conscientious and emotional, and Ravenclaws declared a higher need for cognition. It seems that forced membership allows the detection of at least a few differences that might reflect actual similarities between participants’ traits and house characteristics. If that is the case, forced participants can be described as individuals whose traits (1) may not allow for a clear assignment to a single Hogwarts House (suggesting mixed or borderline membership), which could explain the discrepancy between desired and assigned membership, or (2) may be captured more accurately by the test than by their own self-perceptions or preferences.. On the other hand, differences for the desired memberships are consistent with the house characteristics, which might suggest that the SHQ did not sort them correctly (sort of false membership), that their own choice is a better predictor of their traits than sorting result or, as suggested by Jakob et al. [14], that desire to be in a particular house might influence the way participants answer questions in psychological measures, resulting in mismatch between the SHQ results and their own choices. The fact that the number of participants not sorted to the desired house or desired to be in a different house in the non-reader sample was not enough for the statistical analyses may indicate that the size of the sample was insufficient.

In light of our study results (as well as conclusions made by others – see [13,14]), it is evident that people usually will not get any valid information from SHQ results. Yet, contrary to Crysel et al. [13], we suggest that such results may be more solid for people engaged in particular fiction. For example, recent research indicated that Hogwart House self-identification may be potentially helpful in guiding medical students in choosing a career path [20]. Such potential utilities may be especially beneficial as a part of self-reflection and seem worth noting. Yet, we also feel that despite certain (limited) perks of fandom-based quizzes, they should not be treated too seriously, as they reduce the complexity of psychological functioning into oversimplified variables, such as somebody’s Hogwarts House preference, which tells us little about personal features [21].

While the findings expand psychological theory, our study had some limitations. First, the average age and gender distribution in our sample were not fully representative of the Polish population, i.e., the sample mainly consisted of young (under the age of 30 years) women. Also, participants were recruited using non-probabilistic methods, i.e., snowball online sampling.

The second group of limitations is related to the power of the study, which had an unbalanced design with unequal groups and, therefore, making ANOVA results less trustworthy. Another issue is lower statistical power caused by the small sample sizes, especially in the subsample of non-readers. For instance, in the non-reader subsample, supplemented 95% confidence intervals for effect sizes resulted in relatively wide CIs, which limit the precision of the observed effects. As a consequence, some true effects may have remained undetected, and the strength of the associations in this group should be interpreted with particular caution.

Although unequal group sizes and small samples reduce statistical power, the use of Helmert contrasts minimized the risk of inflated Type I error rates by limiting the number of comparisons per variable. Consequently, the main consequence of this limitation is lower sensitivity to detect small effects in the smaller group, which may have led to some genuine differences remaining undetected. At the same time, the unequal group sizes make direct comparison of effect estimates less straightforward, and thus the results should be interpreted with appropriate caution.

Moreover, although our study offers insights into the relationship between narrative assimilation and personality reporting by including individuals who did not read HP series, the overall sample is dominated by respondents who had certain degree of prior knowledge regarding this series, i.e., readers of the books (subsample 1), and people who did not read the books but mostly saw the movies (subsample 2). Such a limitation was nearly unavoidable with convenience sampling, given the popularity of the HP novels and the resulting difficulty of recruiting individuals who had not read them.

As a result, some of the differences we report between readers and non-readers may reflect differences in statistical power rather than true psychological distinctions. Moreover, because most non-readers were nevertheless familiar with the films, their profiles may be closer to those of readers than to individuals with no exposure to the HP universe, leading to an underestimation of group differences. Still, the fact that most non-readers had some exposure to the films can also be seen as a strength, since it reflects the real-world prevalence of partial familiarity with cultural phenomena of this scale. In this sense, our findings may capture more ecologically valid differences between varying levels of exposure rather than an artificial contrast between “pure” readers and completely unexposed individuals. Future research could nonetheless benefit from including participants with no prior exposure at all, in order to further disentangle the unique contribution of narrative assimilation from background cultural knowledge.

Another limitation is that engagement with the HP fandom was assessed only through questions about reading the books and watching the films. Including items that measured knowledge of the HP universe could have allowed for a clearer distinction between highly engaged and less engaged fans. The last limitation is that the study was non-experimental. This heavily limits our ability to infer the directions of the relationships in which the previously discussed phenomena and variables are involved.

Future studies may address these issues and focus on aspects of experiencing narratives that have not been considered in this study. More variables could also be included in addition to those in the previous studies, especially those which have shown connections to self-perception, like political attitudes [40,41], gaming preferences [42,43], eating behavior [44,45], creativity [46] or cognitive styles [47]. It would help show what types of behaviors and attitudes are influenced by narrative collective assimilation. It would also be worth testing if the results differ for people who first answer the Harry Potter questions and those who begin with the psychological scales. That way, we could verify if focusing and accessing memories about Hogwarts Houses may influence how participants reply to questions about their personality or needs.

## Conclusion

Summing up, in this study, we replicated and broadened the results of previous research concerning the Sorting Hat Quiz in particular by analyzing the possible role of the desired result of this test in predicting differences in psychological traits. The results show that more differences in psychological characteristics specific to members of four Hogwarts Houses were detected based on desired membership and in a group of Harry Potter readers. Moreover, based on analyzing differences for participants not sorted according to their desires, we described our ideas about the Sorting Hat Quiz validity that are worth verifying in future research. The above findings may be useful for researchers developing psychometric tools because they suggest that referencing popular narratives should be avoided for better reliability. Diagnosticians in other fields may find that asking about clients’ favorite narratives and who in those narratives they relate to can show what the ideal self of those people looks like. However, the differences predicted by the SHQ are equally likely to reflect individuals’ engagement with narrative stereotypes and some variations in personality.

## Supporting information

S1 Table Descriptive statistics for Harry Potter fanship variables in three groups of participants. *Note*. ^a^ Reflects questions with some answers omitted because of their impossible or random character.
(DOCX)

S2 Table Frequencies/percentages of answers for Harry Potter fanship questions in three groups of participants. (DOCX)

## References

[1] Gottschall J. The Storytelling Animal: How Stories Make Us Human. Houghton Mifflin Harcourt; 2012.

[2] James E, Mendlesohn F. The Cambridge Companion to Fantasy Literature. Cambridge University Press; 2014.

[3] Brinker F. Chapter 7. Transmedia storytelling in the “Marvel Cinematic Universe” and the logics of convergence-era popular seriality. In: University of Texas Press eBooks. 2017:207–33. doi: 10.7560/312490-009

[4] Green MC. Transportation Into Narrative Worlds: The Role of Prior Knowledge and Perceived Realism. Discourse Processes. 2004;38(2):247–66. doi: 10.1207/s15326950dp3802_5

[5] Hensel WA, Rasco TL. Storytelling as a method for teaching values and attitudes. Acad Med. 1992;67(8):500–4. doi: 10.1097/00001888-199208000-00003 1497776

[6] Smith D, Schlaepfer P, Major K, Dyble M, Page AE, Thompson J, et al. Cooperation and the evolution of hunter-gatherer storytelling. Nat Commun. 2017;8(1):1853. doi: 10.1038/s41467-017-02036-8 29208949 PMC5717173

[7] Stefanek F, Lipka J, Trynduś A. But what doandroids feel about electric sheep? Educating about emotion-related constructs with “Blade Runner”. Studia de Cultura. 2022;14(4):30–44. doi: 10.24917/20837275.14.4.3

[8] Skorupa A, Stefanek F, Paczyńska-Jasińska P. Ciało i umysł w kinie: immersja, doświadczenie emocji i interakcji społecznych podczas seansu w kinie. Studia de Cultura. 1970;13(1):66–78. doi: 10.24917/20837275.13.1.5

[9] Zhang C, Hoel AS, Perkis A. Quality of immersive experience in storytelling: A framework [conference presentation]. 8th International Conference on Quality of Multimedia Experience. Lisbon, Portugal. 2016 June 6–8.

[10] Mikuła E. O tożsamości zjawiska immersji. Czytelnik w dobie “postpapierowej” [On the Identity of the Immersion Phenomenon: The Reader in the “Post-Paper” Era]. In: Urbańska M, ed. Tożsamość [Identity]. Uniwersytet Łódzki; 2018. p. 55–64.

[11] Berberick SN, McAllister MP. Online quizzes as viral, consumption-based identities. Int J Commun. 2016;10:3423–41.

[12] Proudfoot S, Plante C, Reysen S. Why we put on the sorting hat: motivations to take fan personality tests. Current Issues in Personality Psychology. 2019;7(4):265–73. doi: 10.5114/cipp.2020.91473

[13] Crysel LC, Cook CL, Schember TO, Webster GD. Harry Potter and the measures of personality: Extraverted Gryffindors, agreeable Hufflepuffs, clever Ravenclaws, and manipulative Slytherins. Personality and Individual Differences. 2015;83:174–9. doi: 10.1016/j.paid.2015.04.016

[14] Jakob L, Garcia-Garzon E, Jarke H, Dablander F. The Science Behind the Magic? The Relation of the Harry Potter “Sorting Hat Quiz” to Personality and Human Values. Collabra: Psychology. 2019;5(1). doi: 10.1525/collabra.240

[15] Rogoza R, Cieciuch J. Structural Investigation of the Short Dark Triad Questionnaire in Polish Population. Curr Psychol. 2017;38(3):756–63. doi: 10.1007/s12144-017-9653-1

[16] Ashton MC, Lee K. Empirical, theoretical, and practical advantages of the HEXACO model of personality structure. Pers Soc Psychol Rev. 2007;11(2):150–66. doi: 10.1177/1088868306294907 18453460

[17] Dal Cin S, Gibson B, Zanna MP, Shumate R, Fong GT. Smoking in movies, implicit associations of smoking with the self, and intentions to smoke. Psychol Sci. 2007;18(7):559–63. doi: 10.1111/j.1467-9280.2007.01939.x 17614861

[18] Gabriel S, Young AF. Becoming a vampire without being bitten: the narrative collective-assimilation hypothesis. Psychol Sci. 2011;22(8):990–4. doi: 10.1177/0956797611415541 21750250

[19] Djikic M, Oatley K, Zoeterman S, Peterson JB. On Being Moved by Art: How Reading Fiction Transforms the Self. Creativity Research Journal. 2009;21(1):24–9. doi: 10.1080/10400410802633392

[20] Baimas-George M, Vrochides D. The Sorting Hat of Medicine: Why Hufflepuffs Wear Stethoscopes and Slytherins Carry Scalpels. J Surg Educ. 2020;77(4):772–8. doi: 10.1016/j.jsurg.2020.01.004 32033915

[21] Garcia-Garzon E, Jarke H, Jakob L. The Sorting Hat: Cool Fiction Element but Not Necessarily a Good Career Advisor. Collabra: Psychology. 2020;6(1). doi: 10.1525/collabra.16745

[22] Arenas DL, Viduani A, Araujo RB. Therapeutic Use of Role-Playing Game (RPG) in Mental Health: A Scoping Review. Simulation & Gaming. 2022;53(3):285–311. doi: 10.1177/10468781211073720

[23] Scarlet J. Superhero therapy: A hero’s journey through acceptance and commitment therapy. Hachette; 2016.

[24] Matusz PJ, Traczyk J, Gąsiorowska A. Kwestionariusz potrzeby poznania – konstrukcja i weryfikacja empiryczna narzędzia mierzącego motywację poznawczą [Kwestionariusz Potrzeby Poznania – Construction and empirical verification of the scale measuring cognitive motivation]. Psychologia Społeczna. 2011;6(2):113–28.

[25] Cacioppo JT, Petty RE. The need for cognition. Journal of Personality and Social Psychology. 1982;42(1):116–31. doi: 10.1037/0022-3514.42.1.116 7131245

[26] Rogoza R, Rogoza M, Wyszyńska P. Polska adaptacja modelu narcystycznego podziwu i rywalizacji. Polskie Forum Psychologiczne. 2016;21(3):410–31. doi: 10.14656/PFP20160306

[27] Back MD, Küfner ACP, Dufner M, Gerlach TM, Rauthmann JF, Denissen JJA. Narcissistic admiration and rivalry: disentangling the bright and dark sides of narcissism. J Pers Soc Psychol. 2013;105(6):1013–37. doi: 10.1037/a0034431 24128186

[28] Christie R, Geis F. Studies in Machiavellianism. New York: Academic Press; 1970.

[29] Pospiszyl K. Psychopatia [Psychopathy]. Wydawnictwo Akademickie Żak; 2000.

[30] Patrick CJ. Operationalizing the triarchic conceptualization of psychopathy: Preliminary description of brief scales for assessment of boldness, meanness, and disinhibition. Unpublished manual. Department of Psychology, Florida State University, Tallahassee; 2010.

[31] Pilch I, Sanecka E, Hyla M, Atłas K. Polska adaptacja skali TriPM do badania psychopatii w ujęciu triarchicznym. Psychologia Społeczna. 2015;10(4):435–54. doi: 10.7366/1896180020153506

[32] Lee K, Ashton MC. Psychometric Properties of the HEXACO Personality Inventory. Multivariate Behav Res. 2004;39(2):329–58. doi: 10.1207/s15327906mbr3902_8 26804579

[33] Szarota P, Ashton MC, Lee K. Taxonomy and structure of the Polish personality lexicon. Eur J Pers. 2007;21(6):823–52. doi: 10.1002/per.635

[34] Mar RA, Oatley K. The Function of Fiction is the Abstraction and Simulation of Social Experience. Perspect Psychol Sci. 2008;3(3):173–92. doi: 10.1111/j.1745-6924.2008.00073.x 26158934

[35] Cohen J. Audience identification with media characters. In: Bryant J, Vorderer P, editors. Psychology of entertainment. Routledge; 2013. p. 183–97.

[36] Foster JD, McCain JL, Hibberts MF, Brunell AB, Burke Johnson R. The Grandiose Narcissism Scale: A Global and Facet-Level Measure of Grandiose Narcissism. Personality and Individual Differences. 2015;73:12–6. doi: 10.1016/j.paid.2014.08.042

[37] Kaufman SB, Yaden DB, Hyde E, Tsukayama E. The Light vs. Dark Triad of Personality: Contrasting Two Very Different Profiles of Human Nature. Front Psychol. 2019;10:467. doi: 10.3389/fpsyg.2019.00467 30914993 PMC6423069

[38] McCain J, Gentile B, Campbell WK. A Psychological Exploration of Engagement in Geek Culture. PLoS ONE. 2015;10(11):e0142200. doi: 10.1371/journal.pone.0142200 PMC4651513 26580564

[39] Rubin RB, McHugh MP. Development of parasocial interaction relationships. Journal of Broadcasting & Electronic Media. 1987;31(3):279–92. doi: 10.1080/08838158709386664

[40] Feldman S. Values, ideology, and the structure of political attitudes. In: Sears DO, Huddy L, Jervis R, editors. Oxford Handbook of Political Psychology. Oxford University Press; 2003. p. 477–508.

[41] Robinson JP, Shaver PR, Wrightsman LS. Measures of political attitudes. Academic Press; 1999.

[42] Denden M, Tlili A, Essalmi F, Jemni M. Does personality affect students’ perceived preferences for game elements in gamified learning environments? In: 2018 IEEE 18th International Conference on Advanced Learning Technologies (ICALT); 2018. p. 111–5. doi: 10.1109/ICALT.2018.00033

[43] Pyszkowska A, Gąsior T, Stefanek F, Więzik B. Determinants of escapism in adult video gamers with autism spectrum conditions: The role of affect, autistic burnout, and gaming motivation. Computers in Human Behavior. 2023;141:107618. doi: 10.1016/j.chb.2022.107618

[44] Esposito R, Cieri F, di Giannantonio M, Tartaro A. The role of body image and self-perception in anorexia nervosa: The neuroimaging perspective. J Neuropsychol. 2016;12(1):41–52. doi: 10.1111/jnp.12106 27220759

[45] Ullrich NV, Touger-Decker R, O’sullivan-Maillet J, Tepper BJ. PROP taster status and self-perceived food adventurousness influence food preferences. J Am Diet Assoc. 2004;104(4):543–9. doi: 10.1016/j.jada.2004.01.011 15054337

[46] Klebba JM, Tierney P. Advertising Creativity: A Review and Empirical Investigation of External Evaluation, Cognitive Style and Self-Perceptions of Creativity. Journal of Current Issues & Research in Advertising. 1995;17(2):33–52. doi: 10.1080/10641734.1995.10505031

[47] McIntyre RP, Claxton RP, Anselmi K, Wheatley EW. Cognitive Style as an Antecedent to Adaptiveness, Customer Orientation, and Self-Perceived Selling Performance. Journal of Business and Psychology. 2000;15(2):179–96. doi: 10.1023/a:1007775208983

