# Supplementary material for: Harry Potter and personality assessment: The utility of the Sorting Hat Quiz in personality traits’ assessment
Source: PLoS One. 2025 Nov 24;20(11):e0336123. doi: 10.1371/journal.pone.0336123 (PMC12643298; doi:10.1371/journal.pone.0336123)
Supplement: S1 Table — Note. a Reflects questions with some answers omitted because of their impossible or random character. (DOCX) [file pone.0336123.s001.docx]

|  | *N* | *M* | *SD* | Mdn | Min-Max |
| --- | --- | --- | --- | --- | --- |
| **All participants** | | | | | |
| Times of reading books | 676^a^ | 5.15 | 9.08 | 3 | 0-123 |
| Being a fan of books | 677 | 7.14 | 3.22 | 8 | 0-10 |
| Times of watching movies | 673^a^ | 1,690 | 38,740 | 10 | 0-1000000 |
| Being a fan of movies | 677 | 7.45 | 2.51 | 8 | 0-10 |
| Being a fan of the universe | 677 | 5.89 | 3.23 | 7 | 0-10 |
| **Harry Potter readers** | | | | | |
| Times of reading books | 577^a^ | 6.02 | 9.56 | 3 | 1-123 |
| Being a fan of books | 578 | 8.1 | 2.22 | 9 | 0-10 |
| Being a fan of the universe | 578 | 6.44 | 2.91 | 7 | 0-10 |
| **Harry Potter non-readers** | | | | | |
| Times of watching movies | 90 ^a^ | 9.47 | 13.67 | 5 | 1-100 |
| Being a fan of movies | 91 | 5.75 | 2.95 | 6 | 0-10 |
| Being a fan of the universe | 91 | 2.88 | 3.12 | 2 | 0-10 |

**Table S1**

*Descriptive statistics for Harry Potter fanship variables in three groups of participants*

*Note.* ^a^ Reflects questions with some answers omitted because of their impossible or random character.
