# Supplementary material for: Harry Potter and personality assessment: The utility of the Sorting Hat Quiz in personality traits’ assessment
Source: PLoS One. 2025 Nov 24;20(11):e0336123. doi: 10.1371/journal.pone.0336123 (PMC12643298; doi:10.1371/journal.pone.0336123)
Supplement: S2 Table — (DOCX) [file pone.0336123.s002.docx]

**Table S2**

*Frequencies/percentages of answers for Harry Potter fanship questions in three groups of participants*

|  | 0 | 1 | 2 | 3 | 4 | 5 | 6 | 7 | 8 | 9 | ≥ 10 |
| --- | --- | --- | --- | --- | --- | --- | --- | --- | --- | --- | --- |
| **All participants** | | | | | | | | | | | |
| Times of reading books | 99/14.6 | 135/19.9 | 100/14.8 | 81/12 | 35/5.2 | 39/5.8 | 21/3.1 | 36/5.3 | 25/3.7 | 6/0.9 | 100/14.8 |
| Being a fan of books | 64/9.5 | 10/1.5 | 15/2.2 | 20/3 | 20/3 | 38/5.6 | 45/6.6 | 63/9.3 | 91/13.4 | 86/12.7 | 225/33.2 |
| Times of watching movies | 12/1.8 | 25/3.7 | 33/4.9 | 48/7.1 | 33/4.9 | 59/8.7 | 29/4.3 | 28/4.1 | 26/3.8 | 4/0.6 | 380/56.1 |
| Being a fan of movies | 20/3 | 9/1.3 | 9/1.3 | 20/3 | 33/4.9 | 24/3.5 | 63/9.3 | 114/16.8 | 113/16.7 | 93/13.7 | 179/26.4 |
| Being a fan of the universe | 73/10.8 | 29/4.3 | 31/4.6 | 39/5.8 | 33/4.9 | 56/8.3 | 67/9.9 | 93/13.7 | 96/14.2 | 55/8.1 | 105/15.5 |
| **Harry Potter readers** | | | | | | | | | | | |
| Times of reading books | 1/0.2 | 135/23.4 | 100/17.3 | 81/14 | 35/6.1 | 39/6.7 | 21/3.6 | 36/6.2 | 25/4.3 | 6/1 | 99/17.1 |
| Being a fan of books | 5/0.9 | 5/0.9 | 6/1 | 13/2.2 | 18/3.1 | 27/4.7 | 44/7.6 | 60/10.4 | 90/15.6 | 85/14.7 | 225/38.9 |
| Being a fan of the universe | 31/5.4 | 19/3.3 | 24/4.2 | 34/5.9 | 27/4.7 | 49/8.5 | 60/10.4 | 88/15.2 | 91/15.7 | 53/9.2 | 102/17.6 |
| **Harry Potter non-readers** | | | | | | | | | | | |
| Times of watching movies | 1/1.1 | 7/7.7 | 12/13.2 | 15/16.5 | 4/4.4 | 8/8.8 | 5/5.5 | 5/5.5 | 6/6.6 | 2/2.2 | 26/28.6 |
| Being a fan of movies | 7/7.7 | 4/4.4 | 1/1.1 | 11/12.1 | 7/7.7 | 9/9.9 | 9/9.9 | 13/14.3 | 14/15.4 | 6/6.6 | 10/11 |
| Being a fan of the universe | 35/38.5 | 9/9.9 | 7/7.7 | 5/5.5 | 6/6.6 | 7/7.7 | 7/7.7 | 5/5.5 | 5/5.5 | 2/2.2 | 3/3.3 |
